# Detecting and tracking depression through temporal topic modeling of tweets: insights from a 180-day study

**DOI:** 10.1038/s44184-024-00107-5

**Published:** 2024-12-06

**Authors:** Ranganathan Chandrasekaran, Suhas Kotaki, Abhilash Hosaagrahaara Nagaraja

**Affiliations:** 1 Department of Information & Decision Sciences, University of Illinois at Chicago, Chicago, IL USA; 2 Department of Biomedical and Health Information Sciences, University of Illinois at Chicago, Chicago, IL USA

**Keywords:** Anxiety, Depression

## Abstract

Depression affects over 280 million people globally, yet many cases remain undiagnosed or untreated due to stigma and lack of awareness. Social media platforms like X (formerly Twitter) offer a way to monitor and analyze depression markers. This study analyzes Twitter data 90 days before and 90 days after a self-disclosed clinical diagnosis. We gathered 246,637 tweets from 229 diagnosed users. CorEx topic modeling identified seven themes: causes, physical symptoms, mental symptoms, swear words, treatment, coping/support mechanisms, and lifestyle, and conditional logistic regression assessed the odds of these themes occurring post-diagnosis. A control group of healthy users (284,772 tweets) was used to develop and evaluate machine learning classifiers—support vector machines, naive Bayes, and logistic regression—to distinguish between depressed and non-depressed users. Logistic regression and SVM performed best. These findings show the potential of Twitter data for tracking depression and changes in symptoms, coping mechanisms, and treatment use.

## Introduction

Depression as a mental illness has been a great concern worldwide, with more than 280 million individuals affected^1^. In the USA, nearly 21 million people suffer from depression with higher incidence among women and younger individuals^2,3^. Depressed individuals may experience a lack of interest in work or daily activities, sleep disorders, fatigue, inability to focus, recurrent thoughts of death, and an increased risk of suicide. Despite the availability of effective treatments, depression goes undiagnosed and untreated in several cases. Depressed individuals may not seek treatment because they may simply be unaware of the condition or get inhibited by the stigma associated with it. Untreated depression or delay in treatment is associated with poor outcomes including worsened mental health and other conditions such as illicit drug disorders, and criminal and social functioning.

Depression is diagnosed by clinicians through multiple screening tools^4,5^ that use self-reported questionnaires and interviews. Despite their reliability, they have been criticized for the choice of cut-offs used^6,7^, ambiguity in items^8^, limitations in applying cross-cultural settings^9–11^, and biases induced by self-reported data. In addition to the diagnostic instruments, clinicians now have access to substantial amounts of data from social media that provide valuable clues about patients’ health. A number of studies have used social media data and machine learning algorithms to detect depression based on the content and language used^12–18^, emotions expressed^19–21^, and pictures posted^22,23^.

Self-disclosure, defined as “the act of revealing personal information to others”^24^ (p. 2), involves sharing updates, thoughts, experiences, and feelings^25^. For those with mental health issues like depression or anxiety, it serves as a coping mechanism and enhances well-being. Despite risks such as stigmatization and social isolation^26,27^, self-disclosure offers therapeutic benefits, including social support and connectedness^28^. Both verbal and written self-disclosures have therapeutic benefits. Writing about emotions helps create coherent narratives, enhances self-reflection, and improves mental health^29^.

The advent of the internet and the proliferation of social media platforms have opened up new possibilities for rapid, self-disclosure of personal information to a fairly large set of people over the digital networks^14,30^. Self-disclosure in social media platforms can take multiple forms including posts revealing personal information, status updates, revealing personal preferences or opinions, narratives of experiences, or displays of pictures and videos. Studies have documented that individuals disclose information about their mental illnesses in their posts on social media networks such as Facebook^31,32^, X (formerly known as Twitter)^33–35^, and Instagram^23,36^. Such self-disclosures on social media provide individuals with a safe space for expressing themselves, while also providing a sense of community, and offering opportunities for coping and empowerment^37^.

Several studies have examined the self-disclosure of mental illnesses on social media to better understand the extent and nature of information being shared and discussed. These studies have examined the broader themes of discourse, language used in the narrative, timing of disclosures, patterns in the words used, etc. using qualitative content analysis^33,34,38–41^. Collectively, these studies provide evidence of the distinct nature of writing and the language used in social media posts by depressed individuals. Another group of studies has employed advanced machine learning and natural language processing techniques to assess disclosures on social media to detect or predict the risk of depression or other mental illnesses. The focus of these studies has been to unobtrusively observe the user-generated content on social media to assess the onset and nature of mental illness, and build classifiers to distinguish between mentally ill users and healthy ones^14,18,20,42–47^. In addition to social media posts, journal entries have also been used to identify thematic markers of mental health disclosures^48^. Collectively, these studies show that the textual content of the discourse in social media, and personal demographics such as age and gender can facilitate automated detection and classification of individuals with mental illness such as depression. Recent studies have also highlighted the use of social media data to estimate population mental health^49^. Our analysis of the current literature reveals some critical gaps. There is only limited examination of the use of social media data to understand how mental illness progresses over time^50^. The nature of the narrative of depressed users could change over time^51^, and we don’t have much knowledge about the changes that occur in the discourse of depressed users. Specifically, we do not know if the clinical diagnosis of depression and its self-disclosure over social media results in any significant changes in the nature of discourse of the depressed individuals. This study seeks to address these gaps.

This study proposes a novel, temporal analysis approach that involves assessing social media data to detect changes in the content of tweets before and after a depression. We combine text mining techniques with machine learning classifiers to detect and distinguish depressed users, and examine how depression markers change prior to and after clinical diagnosis of depression. Our approach and key contributions can be summarized as follows.

1. Using Twitter data of depressed patients who self-disclose their clinical diagnosis, we propose a novel approach involving temporal analysis of tweets to identify and track changes in depression markers ninety days before and ninety days after disclosure of diagnosis. The temporal assessment addresses a critical gap in current literature and responds to research calls that have been made in this regard^38,50,52,53^.
2. We use CoreX topic modeling to derive a rich set of features that distinguishes depressed users from healthy users. This allows us to identify thematic markers of tweets whose changes precipitate diagnosis, as well as changes that ensue clinical diagnosis of depression. We calculate the odds of an individual tweeting about a particular depression thematic marker (e.g., physical symptoms) over time. Our study provides insights into changes seen in depressed users’ tweets before and after diagnosis.
3. We perform a comparative evaluation of multiple machine learning classifiers to determine an optimal classifier to detect depressed patients from their tweets.

## Methods

### Data collection and user selection

**Fig. 1:**
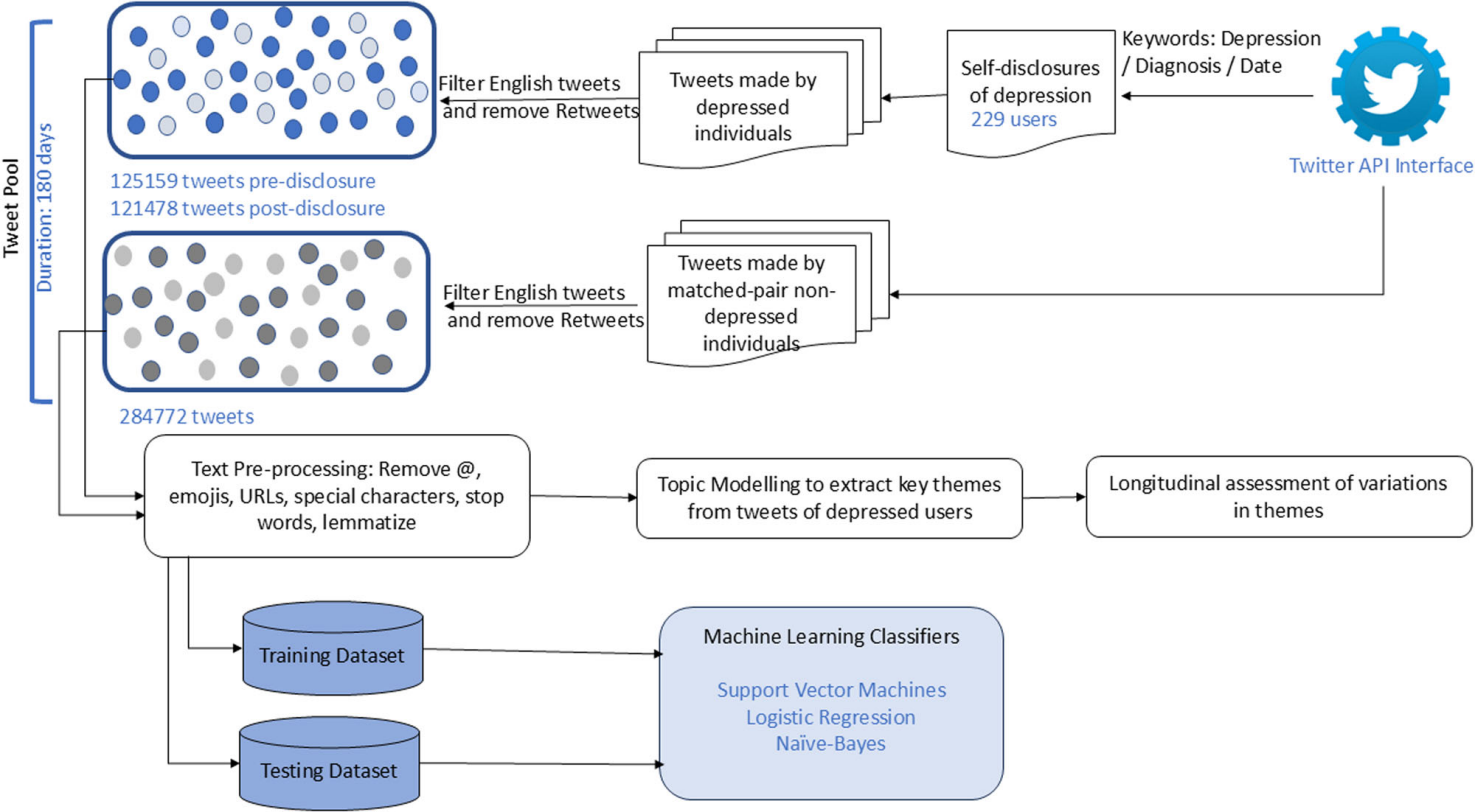
Overview of research methods. Research process illustrating data collection, preprocessing, and analysis using text mining and machine learning classifiers.

Our overall research methodology is shown in Fig. 1. We searched for English-language tweets from Twitter via its public streaming application programming interface, using a set of keywords indicative of self-reported diagnoses of depression. For inclusion, the tweets had to satisfy three criteria. (i) contain keywords and phrases indicating depression (eg. ‘depress’, ‘depression’, ‘major depression’, and its variants), (ii) mention diagnosis (eg. ‘diagnose’, ’assessed’ and variants), and (iii) timestamp indicating the actual date of diagnosis (e.g., today, yesterday, two days ago, etc.). We further checked the tweets to ensure that they contained one of the personal pronouns: I, my, or me, so that the tweets were about themselves. We searched all the tweets that were made in 2019–2020, and filtered 258 users that met our criteria. We further scrutinized the tweets manually and removed duplicates of others’ posts, and those that seemed to be not about self (e.g., “my husband got diagnosed for depression today”).

For each user, based on the diagnosis date, we extracted and examined all the tweets made by each user in the ninety days preceding and succeeding the diagnosis date. Our choice of 180 days surrounding the disclosure date is guided by and consistent with other studies that have examined longitudinal data from social media^54–56^. We, therefore, eliminated users who did not have adequate tweets in the ninety days prior to and after their diagnosis. We removed retweets and retained only the original ones. We obtained the age and gender information of users by searching the tweets made by a user to see mention of age or gender-related information. We removed those users where age and gender could not be determined. Our final dataset contained 229 self-disclosed, clinically diagnosed depressed users who made 246,637 tweets in the 180 days surrounding the diagnosis date. Of the total tweets, 125159 (50.75%) were made prior to clinical diagnosis, and 121,478 (49.25%) were made in the post-diagnosis period. Each day in our examination period saw about 1370 tweets made by depressed users. Table 1 presents the demographics of users in our dataset and Table 2 shows a sample of self-disclosure tweets.

**Table 1:** Demographics of users in the dataset

	*n*	%	Number of tweets prior to depression diagnosis	Number of tweets after depression diagnosis
Age				
≥18	38	16.59	16,070	20,271
19–29	126	55.02	69,520	66,717
30–39	27	11.79	19,943	15,782
≥40	38	16.59	19,626	18,708
Gender				
Male	118	51.53	63,570	56,531
Female	111	48.47	61,589	64,947
Total	229	100	125,159	121,478

**Table 2:** Illustrative tweets disclosing depression diagnosis

*I went to the therapist today. I’ve been diagnosed with depression and was prescribed some antidepressants hopefully I’ll feel human again and not as hollow and unfeeling as before.*
*I finally saw a psychiatrist today and was clinically diagnosed with anxiety, depression, and PTSD. Here’s to hoping meds help me finally get on track!!*
*I was officially diagnosed with severe depression today.*
*I was diagnosed with severe depression and anxiety today. I start therapy In a week and I’m just proud I can finally start working through trauma and healing.*
*Today I got officially diagnosed (finally) with depression anxiety and obsessive-compulsive tendencies. I will start therapy in 2 weeks and see if I can finally improve my life.*
*So I was diagnosed with depression/anxiety today. Kind of figured that would happen at least now I can get it under control.*
*Today I was diagnosed with depression, anxiety w/panic disorder.*

Parallely, we constructed a control group dataset that will allow statistical comparisons between users who self-disclosed their depression and those who did not. For each depressed user, we randomly identified a matched control user, matched by age and gender, and collected tweets made by them in the 180-day period surrounding the disclosure date of the depressed user. Additionally, we ensured choosing a non-depressed matched pair user by checking they did not have any mention of depression-related terms in their tweets. In this way, we compiled the timeline of tweets for a matched control group of users. This resulted in 284,772 tweets made by control group users, averaging 1026 tweets each day.

### Analytical techniques

We were primarily interested in two types of comparisons. First, to compare social media disclosures by depressed users before and after clinical diagnosis. Second, to examine differences in markers between depressed and non-depressed (control group) users.

To perform the temporal analysis of social disclosures prior to and after the clinical diagnosis of depression, we employed a machine learning technique for text mining known as topic modeling to identify hidden topics in Twitter posts. Topic modeling is a useful approach to identifying broader themes in textual data that are not captured by simpler text analysis at the level of words, tokens, and sentences. Topic modeling has been widely used to assess social media data in mental health contexts^57,58^, and topic modeling on notes from electronic health records have been found to be effective in predicting depression^59,60^.

There are several popular topic modeling approaches, including the latent Dirichlet allocation (LDA) algorithm^61^, that utilize probabilistic generative models that speculate the mechanisms for how documents are generated in order to infer latent topics. A relatively new approach, correlation explanation (CorEx) does not assume any underlying domain knowledge and finds coherent, meaningful, hidden topics from the text^62^. CorEx has been increasingly used in several health-related studies to assess social media data^63–66^.

Before performing topic modeling, we cleaned and preprocessed the tweets in two steps. All the words in the tweets were transformed into lowercase, and @mentions, special characters, emojis, and URLs were removed. We also removed stopwords. We then performed lemmatization, a procedure in NLP to identify the root form of inflected words in the English language. spaCy, a NLP library in Python was used for preprocessing and lemmatization.

We implemented the CorEx on our dataset with a different number of topics (*n* = 10, 20, 30, 40, and 50). The specification of the number of topics is important to identify the appropriate latent topics underlying the tweets. Specifying a larger number might produce redundant, irrelevant, or unclear topics. To figure out the optimal number of topics, we computed the distribution of total correlations for each topic to see how much correlation each additional topic contained. This was iteratively done to find the optimal number of topics that could be grouped by the authors into higher-level themes that were meaningful and consistent with our research goals pertaining to depression detection. The correctness of the categorization of topics into themes and keywords was assessed through review by two clinicians who are experts in mental health.

Our next step was to assess the extent to which each tweet was associated with the themes that were discovered from topic modeling. This was done by examining point-wise total correlation (TC) scores provided by CorEx for each of the tweets. CorEx provides a vector of TC values (*log_z)* that indicates the strength of the association of a given tweet with each of the topics. The vector of TC scores indicates the strength of the association of a given tweet with each of the topics extracted. TC scores for each theme were then averaged for each day for all users and corresponding *z*-scores were computed, covering the pre-disclosure and post-disclosure periods. This helped us observe theme-specific changes and variations longitudinally in the periods preceding and succeeding the depression diagnosis.

We used conditional logistic regression to compute the odds of a user tweeting about a specific theme (e.g. mental symptoms) in the post-diagnosis, as compared to the pre-diagnosis period. For instance, what are the odds of an individual user who has disclosed the clinical diagnosis of depression on Twitter to tweet about the causes of depression post-diagnosis, as compared to pre-diagnosis? Analyses were done using the “clogit” command in STATA software.

Finally, to assess differences between depressed and control group users, we built binary classifiers using the TC scores for themes extracted from the previous step as principal features, along with demographic variables age and gender. We implemented three classification algorithms: logistic regression (LR), support vector machines (SVM), and Naïve Bayes (NB) classifiers via ScikitLearn. These techniques have been commonly used in studies using social media for mental health^67^. We used a ten-fold cross-validation wherein the dataset was randomly divided into ten subsets with the same sample size. The performance of classification models was assessed using five metrics: accuracy, precision (number of true positives over the sum of the number of true positives and false positives), recall (number of true positives over the sum of the number of true positives and false negatives), *F*1 measure (tradeoff between precision and recall, captured by their harmonic mean) and recipient operating classification (ROC) curves.

## Results

### Themes from topic modeling

**Fig. 2:**
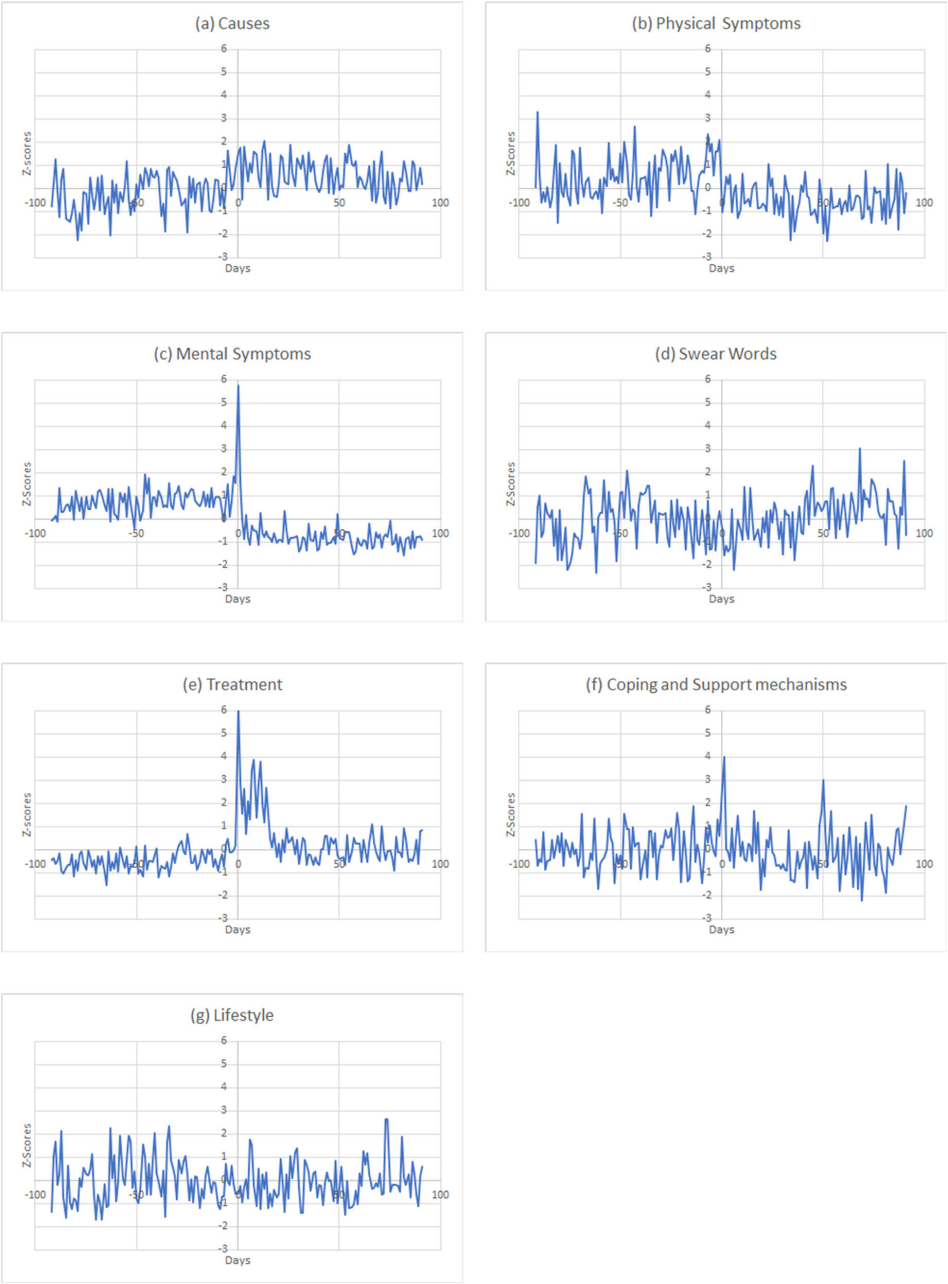
Temporal variations in theme *z*-scores across the pre- and post-diagnosis periods. Each panel depicts the variations in the themes extracted topic modeling of Twitter posts in the 90-day pre-diagnosis period, and 90 days post-diagnosis. Each panel pertains to one of the themes (**a**) causes, **b** physical symptoms, **c** mental symptoms, **d** swear words, **e** treatment, **f** coping and support mechanisms, and **g** lifestyle.

The results from the CorEx topic modeling highlighting the broader themes along with the most contributing keywords are presented in Table 3. The seven themes revolve around the characteristics of depression and are prevalent in both periods before and after the diagnosis disclosure. However, we observe distinct shifts and patterns in the occurrences of these themes in the tweets before and after the diagnosis disclosure. Figure 2 shows the theme-specific variations in *z*-scores across the entire time period of examination.

**Table 3:** Themes and keywords from topic modeling

Themes	Top contributing keywords
Causes	Attack, death, war, suffering, abuse, rape, trauma, accident, assault, harassment, torture, and divorce.
Physical symptoms	Sleep, headache, vomiting, nightmare, pain, migraine, aches, insomnia, vision, and appetite.
Mental symptoms	Cry, sad, panic, stress, upset, anger, worry, mood, suicide, guilt, anxiety, empty, lonely, panic, insecurity, suicide, frustration, disgust, and depression.
Swear words and cursing	Sshit, fuck, bitch, ass, damn, suck, trash, bullshit, asshole, crap, bastard, whore, and goddamn.
Treatment	Doctor, therapy, therapist, hospital, treatment, antidepressant, drug, meds, psychiatrist, psychologist, Zoloft, Prozac, and pill.
Coping and support mechanisms	Exercise, gym, motivate, heal, diet, recovery, hobby, yoga, hope, fitness, run, and support.
Lifestyle	Video, art, music, book, cat, dog, play, event, party, reading, pet, writing, vacation, and gaming.

For the theme pertaining to causes of depression, we find keywords such as attack, accident, trauma, divorce, death, threat, and harassment to characterize this theme. These words capture some of the major causes of depression as expressed by the patients in their tweets. Though the exact causes of depression might differ, past research has documented marital dissolution^68,69^, trauma^70^, intimate partner violence^71^, and bereavement^72^ as some of the common causes of depression. Usage of the keywords in tweets such as, “*I have been traumatized by my brother raping me*”, and “*This whole situation is really making me think of my assault and it is so sad. My assaulter left me in my vomit*” reveal that users in our dataset were engaging in discussing their personal experiences and causes for depression. Figure 2a gives insights into the trends pertaining to this theme in the period prior to and after the depression diagnosis. Depressed users engaged in discourses about causes much more in the post-diagnosis period as compared to the pre-diagnosis phase. Once clinically diagnosed, users seem to reflect on the causes of depression and share this information on social media.

The second theme pertained to physical symptoms experienced by depressed users. Keywords such as headache, vomit, nightmare, sleep, and pain characterize this theme. These keywords pertaining to physical symptoms seem to point out comorbidity of depression with other disorders such as insomnia^73^, migraine^74^, and chronic pain^75^, validating prior studies. Tweets such as “ *I have to get up in 6* *h and it takes me 2* *h to even fall asleep*” and “*Laugh all u want but behold some of the reasons for me to continue living despite being in pain & battling insomnia*” provide pointers to potential comorbid conditions in the depressed users. From Fig. 2b, we note that users reveal physical conditions well before the formal diagnosis of depression. The discussion about physical symptoms decreases after the diagnosis and disclosure.

The third theme relates to the mental conditions of depressed patients. We found several keywords that characterize this theme viz. cry, sad, panic, stress, worry, mood, suicide, guilt, anxious, empty, lonely, etc. Many of these keywords resonate closely with indicators that have long been identified with depression and have been regularly used in depression screening scales like the Patient Health Questionnaire^76^. Our results are consistent with other studies that have identified similar keywords associated with depression^48,58^. Our findings indicate that Twitter posts of depressed users often contained explicit references to their mental conditions that are relevant to the disorder. When we examined the trends of mental conditions over time, we found the *z*-scores associated with the expression of mental symptoms to be relatively more in the pre-diagnosis phase as compared to the post-diagnosis period. Our findings suggest that social media data shows mental symptom markers much before depression is clinically diagnosed, however, the mental symptoms, as expressed in social media, seem to decline in the post-diagnosis period.

Fourth, we detected swearing to be a key theme in the discourse associated with depression. In broader terms, swearing refers to the activity of using taboo words to convey strong emotions^77^. Swearing has been seen as an approach to reduce pain or tension release in individuals.^78^. Depressed users tend to swear in the presence of others^79^ and prior studies have found swearing to be a distinctive marker of language used by depressed users^57,80^. Our topic modeling results showed depressed users commonly use swear words in their Twitter posts. This was prevalent in both time periods preceding and succeeding the clinical diagnosis.

The fifth theme concerns depression treatments. We found disclosing users to share content about their anti-depression medications, side effects, clinic visits, counseling, therapy sessions, and treatment plans. Twitter uses in our dataset shared tweets such as “*I started taking zoloft today*”, “*I got myself a doctors appointment I was diagnosed with severe depression and panic disorder/anxiety and have been put on sertraline (Zoloft) antidepressant*”, “*I’m still depressed and now on Seroquel. And my mouth may permanently taste terrible*.” We also found users to share their experiences with drugs and treatment plans. When we examined the temporal trends about treatments, we found *z*-scores to be higher in the post-diagnosis and disclosure phase as compared to the earlier period.

We labeled the sixth theme as ‘coping and support mechanisms’ based on the associated keywords which included exercise, workout, diet, yoga, gym, heal, hope, support, etc. This theme broadly relates to physical, behavioral, and emotional efforts to deal with stressful events that can eventually lead to or increase depression disorder. We found Twitter users in our dataset to share information about their exercise routines and intentions. For instance, tweets such as “*Started back exercising as part of my mental health recovery”*, and *“Currently trying to gain the courage to go to the gym later today. I’m so sick of the way I look*” indicate the use of exercise as a part of the coping process of some of the depressed users. We found depressed users in our dataset to disclose changes to their diet and food behavior *“I’m going vegan”, I’m trying to avoid fried foods and eat /healthy/”, “I started doing the keto diet around March 10th and I weighed 165 lbs I now weigh 146* *lbs. I’m really proud*.” We also found mentions of yoga by depressed users in many tweets: *“It’s gonna be lots of fun since I’m doing a yoga retreat”, “We just did power yoga and that shit was hella intense”, “Workout done now time to get ready for yoga*”. Trends from Fig. 1f indicate some noticeable changes in the use of coping and support mechanisms after clinical diagnosis, though some of these mechanisms were mentioned in the pre-diagnosis period as well.

The seventh theme was labeled as lifestyle. This theme was characterized by keywords such as pets, dog, cat, video, music, vacation, art, game, event, etc. This theme consisted of tweets that were suggestive of general lifestyle and activities that were pursued by the users such as video gaming, travel, use of pets, etc. Depressed users had tweeted about the helpfulness of pets (e.g., “*Life is hard I just wanna be in bed with my cat*”) and video games (e.g., “*gaming makes me the happiest but it gets lonely*”). We found *z*-scores of the lifestyle theme to be almost similar in both pre- and post-diagnosis periods.

In Table 4, we present the results of the conditional logistic regression and resultant odds ratios pertaining to a user tweeting about a particular depression theme in the post-diagnosis period compared to tweeting about the same theme in the pre-diagnosis period. The odds ratios help us assess the likelihood of the depression theme occurring in the tweets in the post-diagnosis period as compared to the pre-diagnosis period. Our results indicate that depressed users are likely to tweet more about causes, treatment, and coping and support mechanisms in the post-diagnosis period as compared to the pre-diagnosis period. Further, we also find that depressed users are less likely to tweet about physical symptoms, mental symptoms, and the use of swear words in the period following a depression diagnosis. The odds ratio pertaining to lifestyle-related tweets was found to be insignificant.

**Table 4:** Likelihood of tweeting about depression themes pre- and post-diagnosis: conditional logistic regression results

Theme	Odds ratio	95% CI	*p*
Causes	2.22	1.29–3.82	*p* < 0.001
Physical symptoms	0.32	0.14–0.71	*p* < 0.001
Mental symptoms	0.74	0.62–0.89	*p* < 0.001
Swear words	0.73	0.53–1.01	*p* < 0.05
Treatment	3.1	1.71–5.61	*p* < 0.001
Coping and support mechanisms	1.86	1.24–2.81	*p* < 0.001
Lifestyle	1.24	0.85–1.82	Not sig.

### Machine learning classifiers to distinguish depressed users from the control group

In order to validate and establish associations surrounding the themes extracted from the discourse of depressed users, we built three different binary classifiers (SVM, LR, and NB) to compare with our matched control group. We implemented a ten-fold cross-validation routine for the classification task where the entire dataset was divided into ten equally sized subsets, and the classification was performed ten times, each time with a different subset as test data, and all other subsets included as training data. The classification performance metrics for each of the iterations were then averaged. Performance metrics of the binary classifiers are shown in Table 5.

**Table 5:** Performance metrics for classifier algorithms (depressed users vs control group)

Metric	SVM	LR	NB
Accuracy	90.93	90.58	79.71
Recall	92.61	90.61	79.8
Precision	90.2	90.63	80.1
AUC ROC	0.91	0.91	0.8
*F*1-score	91.21	90.58	79.67

The results show that both LR and SVM yielded the best performance with an average accuracy, precision, recall, and *F*-1 score exceeding 90. The good performance of LR (AUC = 0.91) and SVM (AUC = 0.91) is also evident from Table 5. We found that the inclusion of themes from CorEx topic modeling, controlling for age and gender yields better performance as compared to the model that included themes only. Our classifier is able to better detect and distinguish depressed users from their Twitter posts. We also built additional classifiers based on pre-diagnosis tweets of depressed users, and post-diagnosis of depressed users and assessed if they distinguished from the control group, and found acceptable performance of classifiers. However, the performance metrics of classifiers using combined tweets were relatively higher than those that use a subset of pre-diagnosis tweets or post-diagnosis ones.

## Discussion

Analysis of self-disclosures about depression in social media can provide rich insights into the onset and progression of the disease in individuals. By analyzing the content shared around depression on Twitter, this study demonstrates that a more comprehensive view of depression including its causes, potential comorbidities, mental symptoms, experience with treatments, and coping mechanisms can be obtained by mining social media data. By examining a corpus of tweets from 229 depressed users over 180 days using text mining and machine learning techniques, we have noted changes to the social media content posted by depressed users over time. We identified seven themes that serve as markers of depression in the Twitter discourse of depressed users and found distinct differences in these markers in periods preceding and succeeding the date of clinical diagnosis. We found depressed users to likely disclose potential causes of their condition after the clinical diagnosis, whereas the physical and mental symptoms, including comorbidities, start manifesting much before the actual diagnosis. However, in the post-diagnosis period, there are trends from tweets that are indicative of a decrease in negative conditions such as physical and mental symptoms. We found depressed users in our dataset to indicate embracing different coping strategies and support mechanisms, in addition to discussing the efficacies of the clinical treatments.

The themes identified offer interesting insights into how individuals discuss their mental health, particularly in the context of depression on social media. For example, the themes related to *causes* of depression often included trauma, divorce, and loss, with increased discourse on these topics in the post-diagnosis period. This suggests that after receiving a diagnosis and disclosing it on social media, individuals are more likely to reflect on and share the triggers or root causes of their mental health struggles. For instance, one of the users had tweeted “*My parents were getting divorced*”, citing divorce and separation of parents as the reason behind his depression. Another user has indicated the abusive marriage as the main cause of her depression as seen in the tweet “*I got into a marriage that was abusive*”.

The presence of physical symptoms, such as headaches, sleep disturbances, and pain, often appeared in tweets well before the diagnosis, reflecting common comorbidities that have been observed in depression research. Symptoms such as sleeplessness and headaches were mentioned by multiple users as evidenced in the illustrative post: “*I don’t sleep.. I get headaches and severe nightmares*”. Post-diagnosis, the prevalence of such symptoms tended to decrease. This reduction could be due to clinical interventions following the diagnosis. Text mining of users’ social media posts can provide significant insights into the presence of comorbid conditions in depressed users. Identifying such comorbidities could greatly enhance the treatments and clinical interventions provided to depressed patients.

Mental symptoms, including sadness, anxiety, and feelings of emptiness, were also expressed predominantly in the pre-diagnosis phase, indicating that users often experienced significant psychological distress before seeking a formal diagnosis. Tweets such as *“I’m tired of being lonely,” “Feeling anxious all the time,” and “It’s lonely out here… and I don’t get out much”* offer cues about the emotional and psychological distress. These illustrative posts reflect key mental symptoms commonly associated with depression, including persistent feelings of loneliness, isolation, and anxiety. The repetitive nature of these sentiments underscores the depth of their distress, as individuals use social media to articulate their emotional pain and the ongoing challenges in their daily lives. Post-diagnosis, discussions of these symptoms on social media appear to have decreased, perhaps due to therapy or medication.

Interestingly, the use of swear words remained fairly constant before and after diagnosis, which may highlight their use as emotional outlets throughout the depression experience. The themes around *treatment* and *coping mechanisms* reflect individuals’ engagement with healthcare services and self-care practices. Post-diagnosis tweets often included references to antidepressants (e.g., Zoloft, Prozac, Dopemin, etc.), therapy, and side effects of medications, emphasizing the role of social media in allowing users to share their treatment experiences. Sharing treatment information via social media may provide depressed users with a sense of shared identity and support^81^. Meanwhile, tweets about *coping mechanisms* such as exercise, diet, and yoga suggest that users actively seek and implement specific strategies to manage their depression. Engagement in physical activities such as exercise has been documented as an effective mechanism for coping with depression^82,83^. Past research has indicated Yoga^84^ and diet changes^85^ as useful mechanisms to deal with depression. These behaviors, which were mentioned both before and after diagnosis, highlight the importance of self-care in the mental health journey.

The *lifestyle* theme, which includes discussions about pets, music, and video games, remained consistent throughout, suggesting that these elements of everyday life continue to play a role in managing depression, irrespective of the formal diagnosis. Our findings are consistent with prior research where pet ownership^86^ and playing casual video games have been found to exhibit a positive impact on depressed individuals^87,88^.

Some of the major challenges in treating depression relate to non-detection or delayed detection of the disorder, and the lengthy delays between symptom onset and receiving appropriate treatment. We show that mining the social media data can not only provide clues about depression symptoms much before it is formally diagnosed, but also provide a more comprehensive view of the progression of the disease and adjustments being made by depressed patients. We contribute to the extant research by providing a temporal assessment of depression markers over time, specifically by documenting changes in the content of social media posts before and after diagnosis. We provide insights into changes in coping strategies, lifestyle adjustments and treatments that depressed users go through in the time periods prior to and after the clinical diagnosis. Our approach to using themes as primary features to classify depressed users from others yielded performance metrics that are quite comparable and even better than some of the prior studies^14^.

From a methodological perspective, we have combined text mining, machine learning, and statistical modeling to derive rich insights into discourse regarding depression on social media. Our work demonstrates the use of social media data to gain deeper knowledge on multiple themes pertaining to depression and the changes in discourse patterns over time. Additionally, our approach provides a potentially effective way to study depression, offering insights that could be applicable in both clinical practice and community support settings. By identifying changes in depression-related discourse, this method can help mental health professionals and community organizations monitor mental health trends and provide timely interventions, offering an unobtrusive means of supporting individuals dealing with depression.

For health professionals, these findings underscore the importance of monitoring social media for early signs of depression. The temporal changes in themes can provide valuable insights into a patient’s mental state and the effectiveness of treatments. For instance, a decrease in tweets about physical symptoms post-diagnosis may indicate successful management of comorbid conditions, while an increase in discussions about treatment could reflect ongoing engagement with healthcare services. By incorporating social media analysis into clinical practice, health professionals can obtain real-time data to tailor interventions more effectively and support patients throughout their treatment journey. However, it is crucial to ensure that ethical guidelines, including patient consent, privacy protection, and responsible use of personal data, are strictly followed to safeguard individuals’ rights.

Caregivers and family members can also benefit from understanding the online behavior of individuals with depression. Awareness of the themes and their progression can help caregivers provide timely support and intervention. For example, an increase in tweets about coping mechanisms and support networks post-diagnosis suggests a critical period where individuals are actively seeking and benefiting from social support. Encouraging positive interactions and engagement during this time can significantly enhance the well-being of the individual.

On a broader scale, public health initiatives can leverage these insights to design more effective mental health campaigns. The ability to detect and monitor depression through social media offers a scalable and unobtrusive method to reach a larger population. Public health organizations can use this data to identify trends, allocate resources more efficiently, and create targeted interventions that address the specific needs of different demographic groups. Additionally, understanding the stigmatization associated with self-disclosure of mental health issues on social media can inform strategies to reduce stigma and encourage more open conversations about mental health.

This study has several limitations that should be acknowledged. Our analysis relies exclusively on users’ self-disclosures of their mental health conditions on Twitter, which may not be fully representative, as many individuals may choose not to disclose such information or provide only limited details. Moreover, the Twitter user base may not reflect the broader population of individuals with depression, limiting the generalizability of our findings. Additionally, users’ tweet frequency could vary significantly over a period of time, especially, between the pre- and post-diagnosis periods, which may affect the results.

While we documented changes in tweet content before and after diagnosis, we did not assess whether these changes indicated an improvement or worsening of the users’ mental health. Nor did we analyze the quantity, frequency, or specific timing of tweets. Future research could explore whether these factors, along with users’ social networks and tweeting behavior, can provide deeper insights into their mental health trajectories. Further research is needed to assess if the quality of social media posts and posting behavior can be indicative of improvement or worsening of the mental health of depressed users. We also did not consider the user’s social network (e.g., number of followers) and tweeting behavior which could be additional indicators associated with their mental health.

Further, we did not distinguish between different kinds of depression disorders such as bipolar disorder, PTSD, etc. and it is possible that the depression markers vary between them. Although a number of studies have used linguistic variables using tools such as LIWC to detect depression in social media posts with mixed results, we chose to focus on thematic markers. Future studies could also incorporate linguistic, as well as thematic markers, and use Generative AI tools such as GPTs to assess patient-authored social media posts to detect depression from social media posts.

This study demonstrated the effectiveness of text mining and machine learning techniques in detecting and analyzing differences in social media content before and after a depression diagnosis. In addition to detecting potential symptoms, social media data gave more comprehensive information about how depressed users adapt and adjust their behaviors over a period of time. Our findings highlight the potential of using social media data to detect shifts in tweet content, offering valuable clues for real-time identification and monitoring of mental health issues. These insights provide a foundation for providing targeted clinical interventions and tailored support to individuals suffering from depression.

## Data Availability

The dataset, containing the tweet-ids of posts by depressed users, used for this study can be obtained from the corresponding author on request.
